# An Interferon Signature Discriminates Pneumococcal From Staphylococcal Pneumonia

**DOI:** 10.3389/fimmu.2018.01424

**Published:** 2018-06-25

**Authors:** Anja Strehlitz, Oliver Goldmann, Marina C. Pils, Frank Pessler, Eva Medina

**Affiliations:** ^1^Infection Immunology Research Group, Helmholtz Centre for Infection Research, Braunschweig, Germany; ^2^Mouse Pathology, Helmholtz Centre for Infection Research, Braunschweig, Germany; ^3^Institute for Experimental Infection Research, TWINCORE Center for Experimental and Clinical Infection Research, Hannover, Germany

**Keywords:** pneumonia, *Streptococcus pneumoniae*, *Staphylococcus aureus*, biomarkers, interferon, transcriptome

## Abstract

*Streptococcus pneumoniae* is the most common cause of community-acquired pneumonia (CAP). Despite the low prevalence of CAP caused by methicillin-resistant *Staphylococcus aureus* (MRSA), CAP patients often receive empirical antibiotic therapy providing coverage for MRSA such as vancomycin or linezolid. An early differentiation between *S. pneumoniae* and *S. aureus* pneumonia can help to reduce the use of unnecessary antibiotics. The objective of this study was to identify candidate biomarkers that can discriminate pneumococcal from staphylococcal pneumonia. A genome-wide transcriptional analysis of lung and peripheral blood performed in murine models of *S. pneumoniae* and *S. aureus* lung infection identified an interferon signature specifically associated with *S. pneumoniae* infection. Prediction models built using a support vector machine and Monte Carlo cross-validation, identified the combination of the interferon-induced chemokines CXCL9 and CXCL10 serum concentrations as the set of biomarkers with best sensitivity, specificity, and predictive power that enabled an accurate discrimination between *S. pneumoniae* and *S. aureus* pneumonia. The predictive performance of these biomarkers was further validated in an independent cohort of mice. This study highlights the potential of serum CXCL9 and CXCL10 biomarkers as an adjunctive diagnostic tool that could facilitate prompt and correct pathogen-targeted therapy in CAP patients.

## Introduction

Community-acquired pneumonia (CAP) remains a leading cause of morbidity and mortality in both industrialized and developing countries (1). Prompt identification of the causative pathogen and accurate antimicrobial therapy are critical for a favorable outcome (2, 3). However, establishing a microbial diagnosis for patients with pneumonia is still challenging and, despite the rapid development of molecular diagnostic techniques, the causative agent remains unidentified in approximately 50% of CAP patients (4).

Although *Streptococcus pneumoniae* is the most common pathogen causing CAP (5), a wide array of pathogens including viruses can also cause pneumonia (6, 7). In recent years, several studies have reported the occurrence of very severe CAP cases caused by methicillin-resistant *Staphylococcus aureus* (MRSA) (8–10). Because the clinical symptoms of *S. pneumoniae* and *S. aureus* CAP are similar if not identical (11, 12), empiric treatment regimens providing coverage for MRSA such as vancomycin or linezolid are frequently used for CAP patients until a microbiological diagnosis is available (12). This results in the overuse of these antibiotics, which is unfortunate in the light of the current crisis in antibiotic resistance. For this reason, de-escalation of antibiotic therapy from broad-spectrum empiric coverage to narrower spectrum pathogen-targeted therapy has been proposed (13, 14). Key to the success of this approach is the prompt and correct identification of the CAP causative organism.

The gold standard in conventional microbial diagnosis in CAP patients primarily relies on the quantification of microorganisms in culture of respiratory specimens such as sputum, tracheal aspiration, or broncho-alveolar lavage (15). These conventional methods are not only time consuming (48–72 h) but also the low yield is often insufficient for etiological diagnosis and the results can be influenced by previous antibiotic uptake (16). Although new molecular methods such as detection of *S. pneumoniae* antigens in urine (17, 18) and PCR-based assays (19) have been developed, detection of pneumococcal proteins or DNA in CAP patients does not necessarily indicate that this pathogen is the causative organism since *S. pneumoniae* is often carried asymptomatically in the nasopharynx of children and adults (20). Consequently, more accurate diagnostic tools are needed to improve the management of CAP patients that can facilitate pathogen-specific antimicrobial therapy rather than empiric selection of antimicrobial agents with broader spectrum. In this regard, host biomarkers have been proposed as promising tools for diagnosis of CAP (21). The use of biomarkers comprising well-characterized sets of indicators, identified with proteomic, transcriptomic, or metabolomic methods, has been envisaged as an innovative approach to achieve a rapid diagnosis and etiology for several infections including pneumonia (22–25).

The objective of this study was to identify novel candidate host biomarkers to discriminate between *S. pneumoniae* and *S. aureus* pneumonia. We used for this purpose experimental murine models of bacterial pneumonia because they share many clinical and pathophysiological features with human disease. The results of this study have identified the combination of CXCL9 and CXCL10 serum levels as accurate biomarkers to discriminate between *S. pneumoniae* and *S. aureus* pneumonia. These biomarkers can be used in combination with molecular detection for achieving a more accurate etiologic diagnosis of CAP.

## Materials and Methods

### Bacterial Strains

The *S. pneumoniae* strain TIGR4 of the serotype 4 (26) was grown at 37°C in Todd-Hewitt broth supplemented with 1% (w/v) yeast extract and 1% of heat-inactivated fetal calf serum and the MRSA strain USA300 (27) as well as the strain 6850 (ATCC 53657) was grown overnight at 37°C in Tryptic Soy broth. Bacteria were grown to mid-log phase, washed and diluted to the required concentration.

### Mice and Infection Model

Specific-pathogen-free 10- to 12-weeks-old BALB/c and C57BL/6 mice were purchased from Envigo (Horst, Netherlands). The transgenic IFN-β-luciferase reporter mice (Δβ-luc) (28) were bred at the animal facility of the Helmholtz Centre for Infection Research, Braunschweig. For intranasal infection, mice were anesthetized by intraperitoneal injection with ketamine-xylazine solution and inoculated intranasally with either 5 × 10^7^ CFU of *S. pneumoniae* TIGR4 or 5 × 10^8^ CFU of *S. aureus* both in 20 µl of PBS. Mice were euthanized by carbon dioxide asphyxiation at 24 or 48 h of infection and bacterial loads were determined in lungs and liver by preparing homogenates in 5 ml PBS and plating 10-fold serial dilutions on blood agar plates. Colonies were counted after overnight incubation at 37°C.

Animal studies were performed in strict accordance with the German animal welfare regulations and the recommendations of the Society for Laboratory Animal Science (GV-Solas). All experiments were approved by the Niedersächsisches Landesamt für Verbraucherschutz und Lebensmittelsicherheit, Oldenburg, Germany (Permit No. 33.19-42502-04-13/1260 and 33.9-42502-04-13/1195).

### Histopathology

Histological slides of approximately 3 µm thickness were prepared from formalin-fixed, paraffin-embedded lung tissue obtained from infected or PBS-treated mice at 48 h of infection. Sections were stained with hematoxylin and eosin according to standard laboratory procedures and evaluated with a Zeiss Axioscope light microscope, randomized, and blinded to the experimental groups.

### *In Vivo* Imaging

IFN-β-luciferase reporter mice were injected i.p. with 150 mg/kg of d-luciferin in PBS (Calipers), anesthetized using Isoflurane (Baxter) and monitored using an IVIS 200 imaging system (Calipers). Photon flux was quantified using the Living Image 3.0 software (Calipers).

### RNA Extraction of Lung Tissue and Blood

RNA was isolated from lung tissue samples using the miRNeasy Mini Kit (QIAGEN) following the manufacturer’s instructions. Similar amounts of RNA extracted from two to three mice per experiment were pooled. RNA was isolated from the blood samples using the RNeasy Protect Animal Blood Kit (QIAGEN) according to the manufacturer’s instructions. RNA extracted from three to six mice per experiment was pooled. RNA samples were cleared from globin mRNA and rRNA before RNA sequencing (RNA-Seq) using the Globin-Zero Gold Kit (Epicentre). A total of three RNA pools (biological replicates) for each condition and time point were generated. RNA integrity and concentration were determined with Agilent 2100 Bioanalyzer and Nanodrop 1000 spectrophotometer, respectively.

### Library Generation and Sequencing

Libraries from lung tissue and blood RNA were generated using the ScriptSeq v2 RNA-Seq library preparation kit (Illumina) according to the manufacturer’s instructions. Libraries were sequenced on the Illumina HiSeq 2500 platform using the TruSeq S.R. cluster kit, v3-cBot-HS (Illumina). Three to four libraries were multiplexed per lane and sequenced to 58 cycles in one direction. The quality of the raw sequence data was checked by FastQC (389). The RNA-Seq was performed by the Genome Analytics Platform at the Helmholtz Centre for Infection Research in Braunschweig, Germany.

### RNA-Seq Data Processing

Sequence reads of RNA-Seq were preprocessed for quality and trimmed of adapters using fastq-mcf with the following parameters: -q 20, -max-ns 0. RNA-Seq sequence reads were mapped against the GRCm38 genome of ENSEMBLE with the RNA-Seq aligner STAR (390) allowing a maximum of two mismatches, a minimum match length of 18 bp and an intron range between 50 to 15,000. Unique mapped reads were counted using htseq-count (391).

### Real-Time PCR (RT-PCR)

Total RNA was reverse transcribed and amplified using a SensiFAST SYBR No-ROX onestep kit and the Rotor-Gene Q real-time PCR system (Thermofisher). Thermal cycling conditions consisted of reverse transcription for 15 min at 45°C and activation of polymerase for 5 min at 95°C, followed by 40 cycles of 20 s at 95°C, 20 s at 60°C, and 20 s at 72°C. All Primer sequences (see Table S1 in Supplementary Material) were designed to flank a long intron to exclude genomic DNA amplification. Cycle threshold values for each gene were normalized to the housekeeping gene β*-actin*. The relative copy number for each gene was calculated using the Pfaffl equation (29) and expressed as relative mRNA expression in infected animals to that in PBS controls.

### Determination of Cytokines and Chemokines Serum Levels

The concentration of cytokines/chemokines in serum was determined using the bead-based multiplex immunoassay Legendplex™ (BioLegend) according to the manufacturer’s protocol.

### Bioinformatics and Statistical Analysis

Statistical analysis was carried out using GraphPad Prism 5.01 (GraphPad Software, San Diego, CA, USA), PRIMER (v.6.1.6, PRIMER-E; Plymouth Marine Laboratory), MetaboAnalyst 3.0 (30) and the free software R v3.1.3 (http://www.rproject.org). Comparisons between groups or time points were made using Student *t*-test or one-way ANOVA. Principal component analysis (PCA) was conducted using the function prcomp from the R package stats. In addition, the factor loading of each gene for the different principal components was extracted. Differentially expressed genes between conditions were determined using the Bioconductor package DESeq2 implemented in R assuming a negative binominal distribution. A gene was considered significantly different in expression if the adjusted *p*-value was below 0.01. Infection classification models were generated using support vector machine (SVM). To determine the performance of each generated model, a receiver operating characteristic (ROC) curves was carried out and the area under the receiver operating characteristics curve (AUC) was calculated. AUC close to 1 indicates a successful classification model. External cross-validation of the best classification models was determined by Monte Carlo cross-validation (MCCV) method.

Complete sequencing data have been deposited in ArrayExpress: http://www.ebi.ac.uk/arrayexpress/experiments/E-MTAB-5815.

## Results

### Experimental Models of *S. pneumoniae* and *S. aureus* Lung Infection

**Figure 1:**
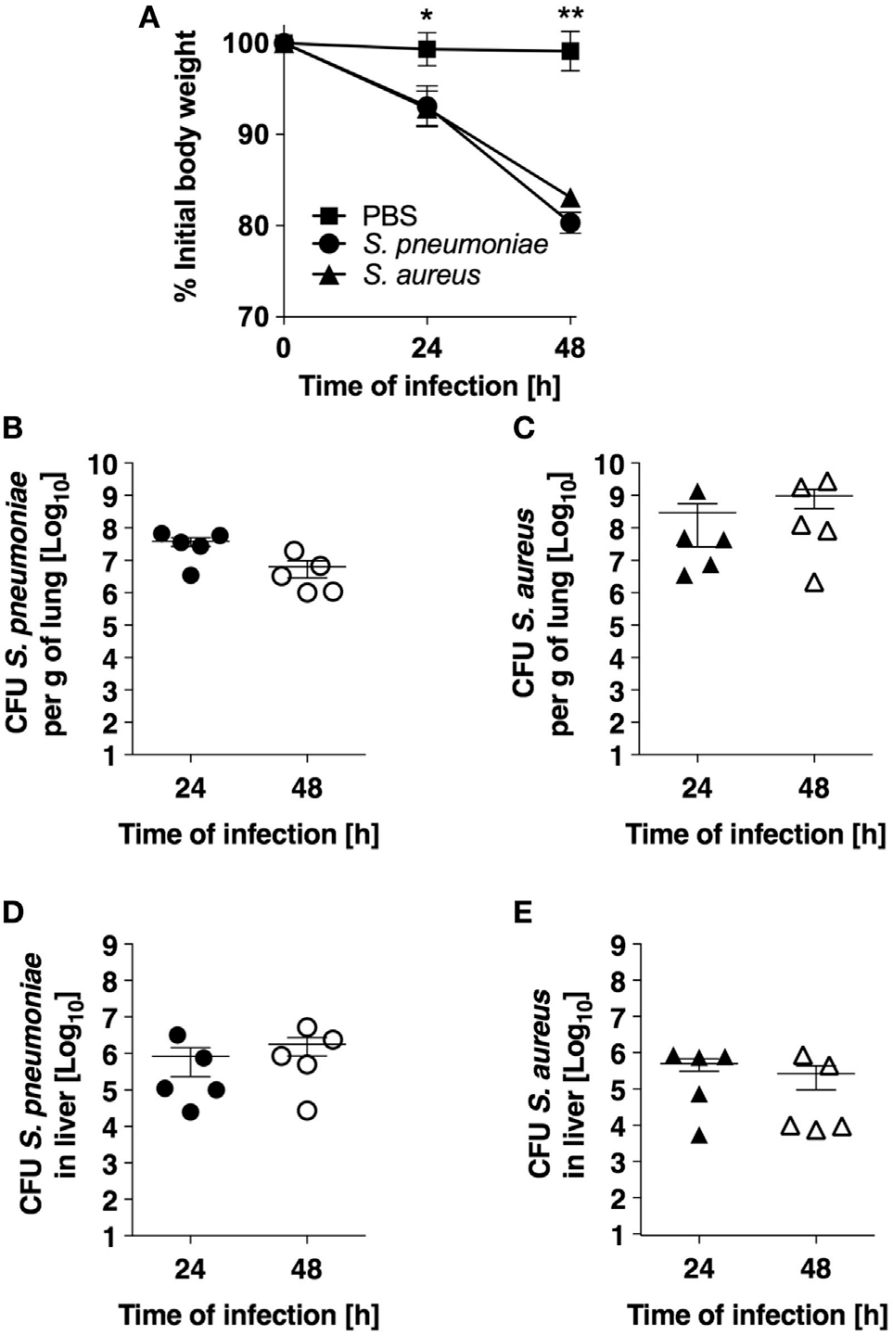
Infection parameters of pneumococcal and staphylococcal pneumonia in experimental murine models. **(A)** Body weight of BALB/c mice intranasally inoculated with either *Streptococcus pneumoniae* (circles) or *Staphylococcus aureus* (triangles). Mice intranasally treated with PBS were used as control (squares). Each symbol represents the mean value ± SD of 5–12 mice. *p < 0.05; **p < 0.01. **(B)** Pulmonary bacterial loads in BALB/c mice after intranasal inoculation with *S. pneumoniae*. **(C)** Pulmonary bacterial loads in BALB/c mice after intranasal inoculation with *S. aureus*. **(D)** Bacterial loads in the liver of *S. pneumoniae*-infected mice. **(E)** Bacterial loads in the liver of *S. aureus*-infected mice. Each symbol represents an individual animal. One representative experiment out of three performed independently is shown.

**Figure 2:**
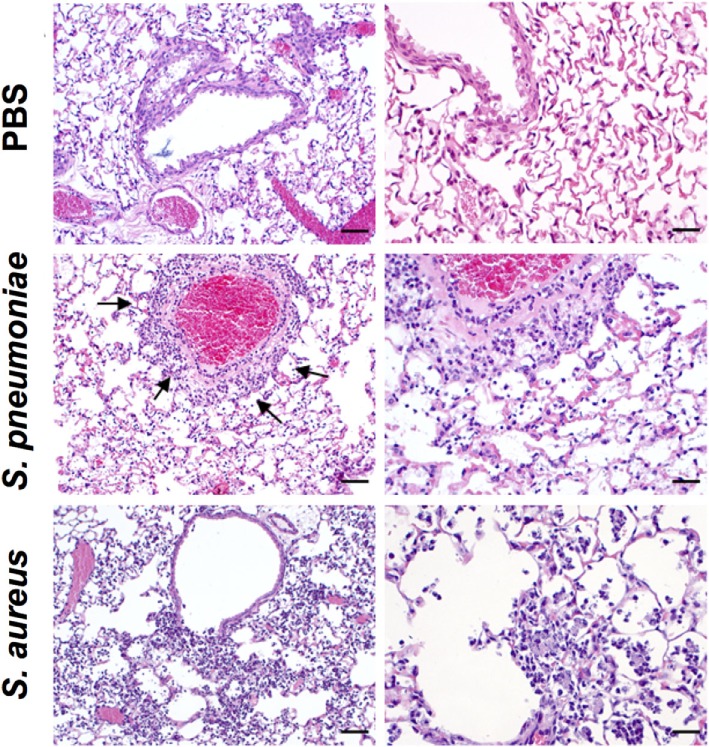
Histopathology (hematoxylin and eosin) of lung tissue obtained from BALB/c mice intranasally inoculated with either *Streptococcus pneumoniae* (middle panels) or *Staphylococcus aureus* (lower panels) or with only PBS (upper panels) at 24 h after treatment. Black arrows indicate peribronchial cellular infiltration. Lower magnification images are shown in the left panels and higher magnification images in the right panels. Original magnification: bar indicates 50 µm for left panels and 25 µm for right panels.

The experimental models of *S. pneumoniae* and *S. aureus* lung infection used in this study consisted in the intranasal inoculation of BALB/c mice with either 5 × 10^7^ *S. pneumoniae* TIGR4 (pneumococcal pneumonia) or with 5 × 10^8^ *S. aureus* USA300 (staphylococcal pneumonia) and mirrored many aspects of the human disease. Both infections induced significant morbidity as shown by the prominent and progressive body weight loss observed in infected mice (Figure 1A). Pulmonary bacterial loads were comparable between *S. pneumoniae*-infected (Figure 1B) and *S. aureus*-infected (Figure 1C) mice and extrapulmonary bacteria dissemination to systemic organs was also observed in both infection groups (Figures 1D,E). Histological evaluation of lung tissue indicated distinctive pathology. While *S. pneumoniae* infection caused a moderate peribronchial and perivascular invasion of predominantly neutrophils, accompanied by some lymphocytes (Figure 2, middle-left panel, black arrows) with moderate to large numbers of bacteria and phagocytic cells present in alveoli (Figure 2, middle-right panel), *S. aureus* induced multifocal alveolar inflammation with large numbers of bacteria visible in the alveoli (Figure 2, lower panels).

### Transcriptional Response in Infected Lungs

**Figure 3:**
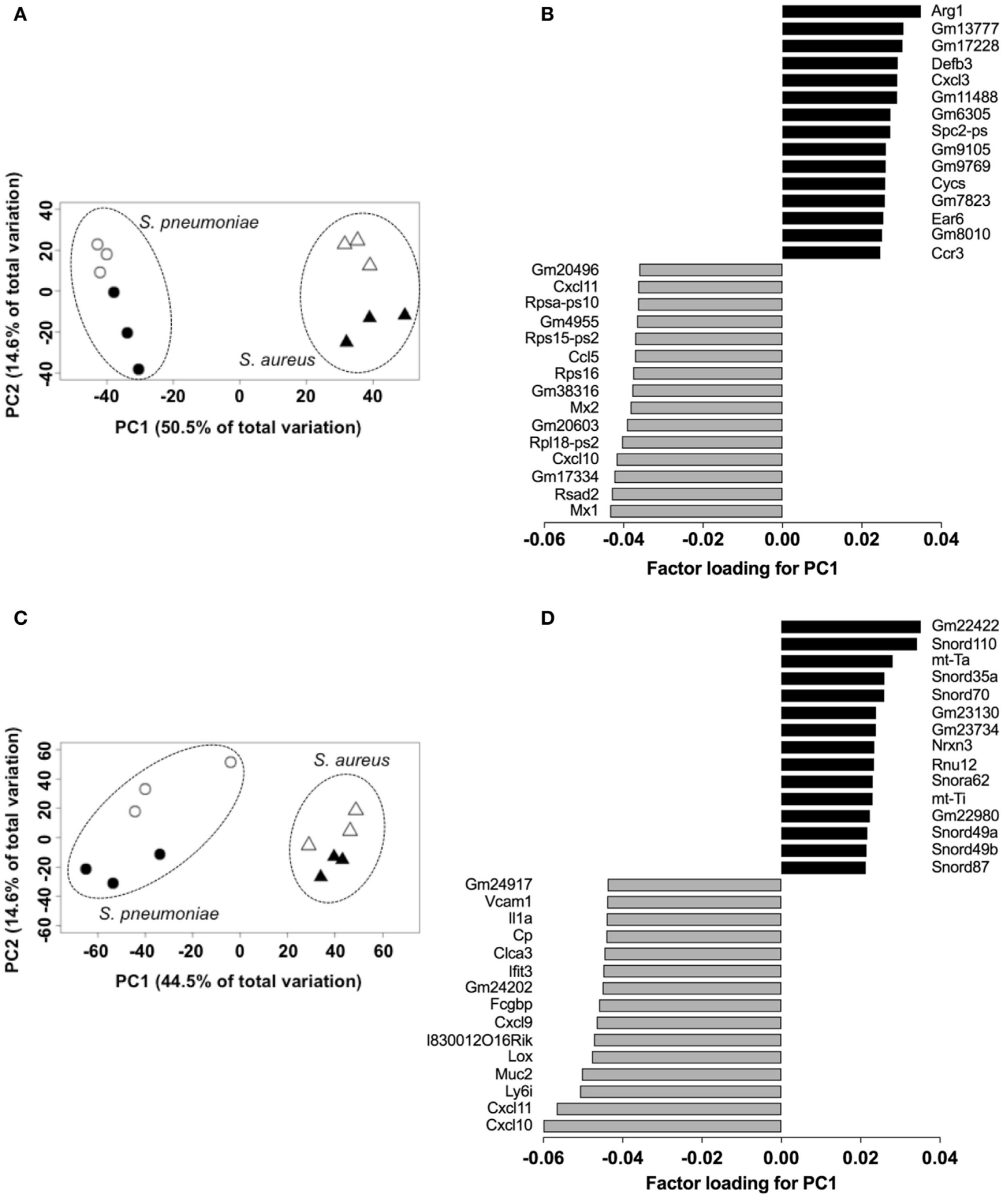
Pathogen-specific transcriptional signatures in lung tissue and peripheral whole blood of infected mice. Principal component analysis of the transcription data of *Streptococcus pneumoniae*-infected (circles) and *Staphylococcus aureus*-infected (triangles) lungs at 24 h (open symbols) and 48 h (solid symbols) of infection in lungs **(A)** and blood **(C)**. Each symbol represents one independent experiment. Factor loading for PC1 analysis of lungs **(B)** and blood **(D)** transcription data. Black bars represent genes expressed to a greater extent in *S. aureus*-infected samples whereas gray bars represent genes expressed to a greater extent in *S. pneumoniae*-infected samples.

In order to establish the proof-of-concept that *S. pneumoniae* and *S. aureus* induced specific transcriptional signature in the infected tissue despite their similar infection outcome, the transcriptional response induced by *S. pneumoniae* and *S. aureus* in the lungs was determined using RNA-Seq and the genes differentially expressed in response to these infections agents were identified using the DESeq2 algorithm. The results revealed that both *S. pneumoniae* and *S. aureus* induced a large shift in gene expression in the lungs respect to uninfected sham-treated controls (see Figure S1 in Supplementary Material). In order to identify pathogen-specific transcriptional signatures in the infected lungs, unsupervised PCA was conducted by entering gene expression data from the different samples without labeling of groups. When the data of all *S. pneumoniae* and *S. aureus*-infected samples from 24 and 48 h of infection were entered, the first principal component (PC1), which explained 50.5% of total variance, separated the 12 samples into two distinct groups that corresponded to *S. aureus* and *S. pneumoniae* infections, respectively (Figure 3A). Among the top genes identified by factor loading for PC1 with greater expression in *S. aureus*-infected lungs (positive values) were *Arg1, Defb3, Cxcl3, Ccr3, Cycs*, and *Ear6* (Figure 3B), whereas mostly interferon-induced genes including *Mx1, Rsad2, Cxcl10, Mx2*, and *Cxcl11* were more highly expressed in the *S. pneumoniae*-infected samples (Figure 3B). These results were validated by RT-PCR using a set of the genes identified by the factor loading for PC1 that discriminated between *S. pneumoniae* and *S. aureus* infection groups (see Figure S2 in Supplementary Material). Thus, *S. pneumoniae* and *S. aureus* induced a distinctive transcriptional response in the lungs.

### Transcriptional Response of Peripheral Blood

**Figure 4:**
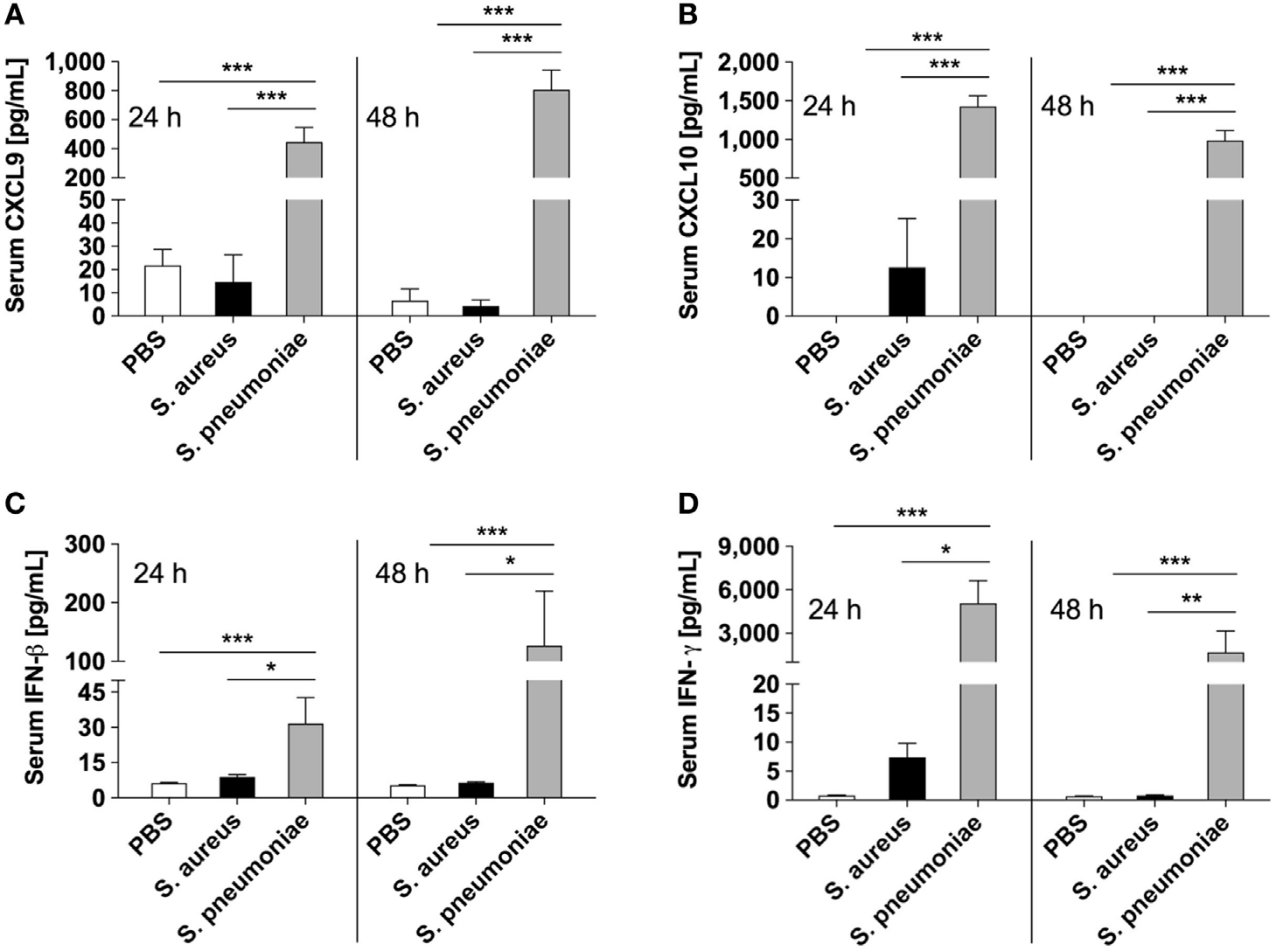
Circulating levels of interferons and interferon-induced mediators in infected mice. Serum levels of CXCL9 **(A)**, CXCL10 **(B)**, IFN-β **(C)**, and IFN-γ **(D)** in *Streptococcus pneumoniae*-infected (gray bars), *Staphylococcus aureus*-infected (black bars), or PBS-treated (white bars) mice measured with bead-based multiplex immunoassay at 24 and 48 h after intranasal inoculation. Each bar represents the mean ± SD of values obtained from eight to nine mice from three independent experiments. *p < 0.05; **p < 0.01; ***p < 0.001.

**Figure 5:**
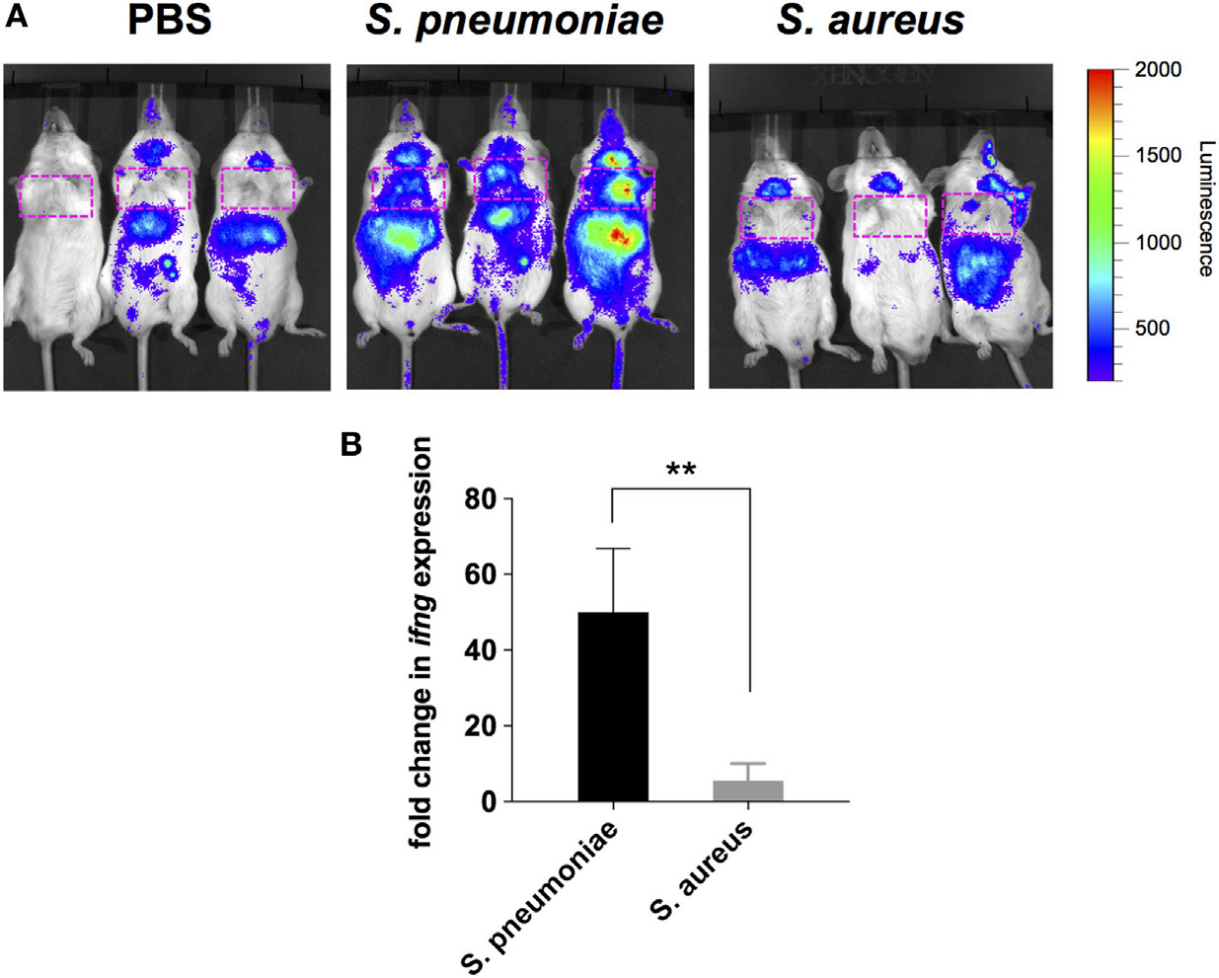
Induction of IFN-β and IFN-γ gene expression in infected mice. **(A)** Luciferase gene expression in Δβ-luc reporter mice infected with *Streptococcus pneumoniae* (middle panel), *Staphylococcus aureus* (right panel), or treated with PBS (left panel) at 12 h post-inoculation. Mice were injected with luciferin (i.p.) and luciferase activity was visualized immediately in the IVIS 200 imaging system. The intensity of emission is represented as a pseudocolor image. The red box indicates the anatomical location of the lungs. **(B)** Quantification of IFN-γ gene expression in the lungs of *S. pneumoniae*-infected (black bar) or *S. aureus*-infected (gray bar) mice at 12 h post-inoculation by real-time PCR. Values are expressed as the fold change between the expression of *infg* in infected mice and the expression of this gene in uninfected control mice. Each bar represents the mean ± SD of three independent experiments. **p < 0.01.

Because peripheral blood cells are more easily accessible than tissue biopsies, they are currently widely used as a surrogate tissue to monitor for disease biomarkers (31). Therefore, we evaluated if the gene expression profile of peripheral whole blood from mice infected with *S. pneumoniae* or *S. aureus* reflected the transcriptional response observed in the lungs. Differential gene expression analysis of peripheral blood from *S. pneumoniae* or *S. aureus*-infected mice taken at 24 and 48 h of infection indicated that both pathogens induced extensive alteration in gene expression compared to uninfected sham-treated controls (see Figure S3 in Supplementary Material). To determine the blood-derived transcriptional signature specific for each pathogen, the gene expression datasets were subjected to unsupervised PCA analysis. Again, PC1, which explained 44.4% of total variation, separated the 12 samples into two distinct groups that corresponded to *S. aureus* and *S. pneumoniae* infection (Figure 3C). In *S. aureus*-infected mice, the blood transcriptional signature did not mirror the transcriptional signature of the lungs since factor loading of PC1 showed greater expression of various small nucleolar RNAs, mitochondrially encoded RNA genes, *Nrxn3* encoding a neuronal cell surface protein, and small nuclear RNA U12 (*Rnu12*) (Figure 3D). By contrast, the genes exhibiting greater expression in *S. pneumoniae*-infected mice were again mostly interferon-induced genes including *Cxcl10, Cxcl11, Ifit3*, and *Cxcl9*, which was completely consistent with the transcriptional profile of lung tissue (Figure 3D). The greater expression of interferon-induced genes in *S. pneumoniae* infection was also reflected at the protein level as shown by the significantly higher amounts of interferon-induced CXCL9 (Figure 4A) and CXCL10 (Figure 4B) in serum of *S. pneumonia*-infected mice in comparison with *S. aureus*-infected or PBS-treated mice. The fact that interferon-induced genes were more highly expressed in *S. pneumoniae*-infected than in *S. aureus*-infected mice suggested that the expression of interferons might also differ between the two groups of infected mice. Indeed, serum levels of IFN-β (Figure 4C) and IFN-γ (Figure 4D) were also significantly greater in *S. pneumoniae*-infected than in *S. aureus*-infected mice. The reason why the genes encoding IFN-β and IFN-γ did not appear in any of the transcriptional analysis that we performed at 24 and 48 h of infection was that these genes were induced by *S. pneumoniae* very early during infection, i.e., before the 24 h time point. Thus, using IFN-β Δβ-luc reporter mice (28), we found that the gene encoding IFN-β was induced by *S. pneumoniae* as early as 12 h post-inoculation (Figure 5A). Similarly, significant upregulation of the gene encoding IFN-γ by *S. pneumoniae* was evidenced by RT-PCR at 12 h of infection (Figure 5B). The failure of *S. aureus* to elicit an interferon response in mice after intranasal administration was independent of the *S. aureus* strain used for infection as demonstrated by the lack of interferon response in the circulation of mice intranasally infected with the methicillin-sensitive *S. aureus* strain 6850 (data not shown).

Together these results confirmed that *S. pneumoniae* induced a significantly more vigorous interferon-related response in lungs and peripheral blood than *S. aureus* after respiratory inoculation in mice.

### Performance Evaluation of Interferon-Related Biomarkers for Discriminating Pneumococcal From Staphylococcal Pneumonia

**Figure 6:**
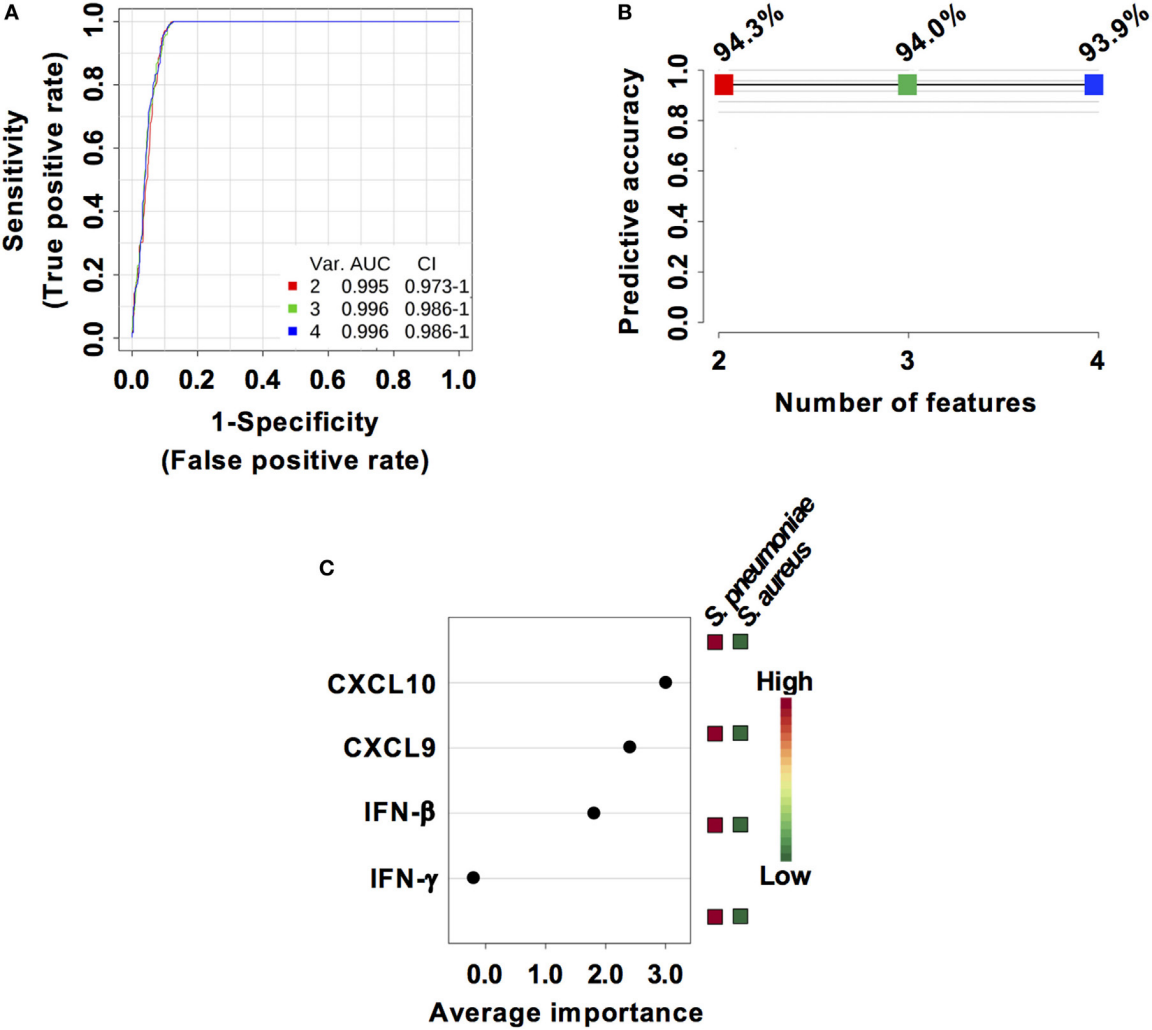
Predictive models based on IFN-β, IFN-γ, CXCL9, and CXCL10 serum levels built by support vector machine (SVM). **(A)** Receiver operating characteristic curves and area under the receiver operating characteristics curve (AUC) values of the predictive classifiers built on 2, 3, and 4 features using an SVM. **(B)** Predictive accuracy of the different predictive models. **(C)** Average importance of each of the four features included in the predictive models and abundance in *Streptococcus pneumoniae* and *Staphylococcus aureus* infection samples.

**Figure 7:**
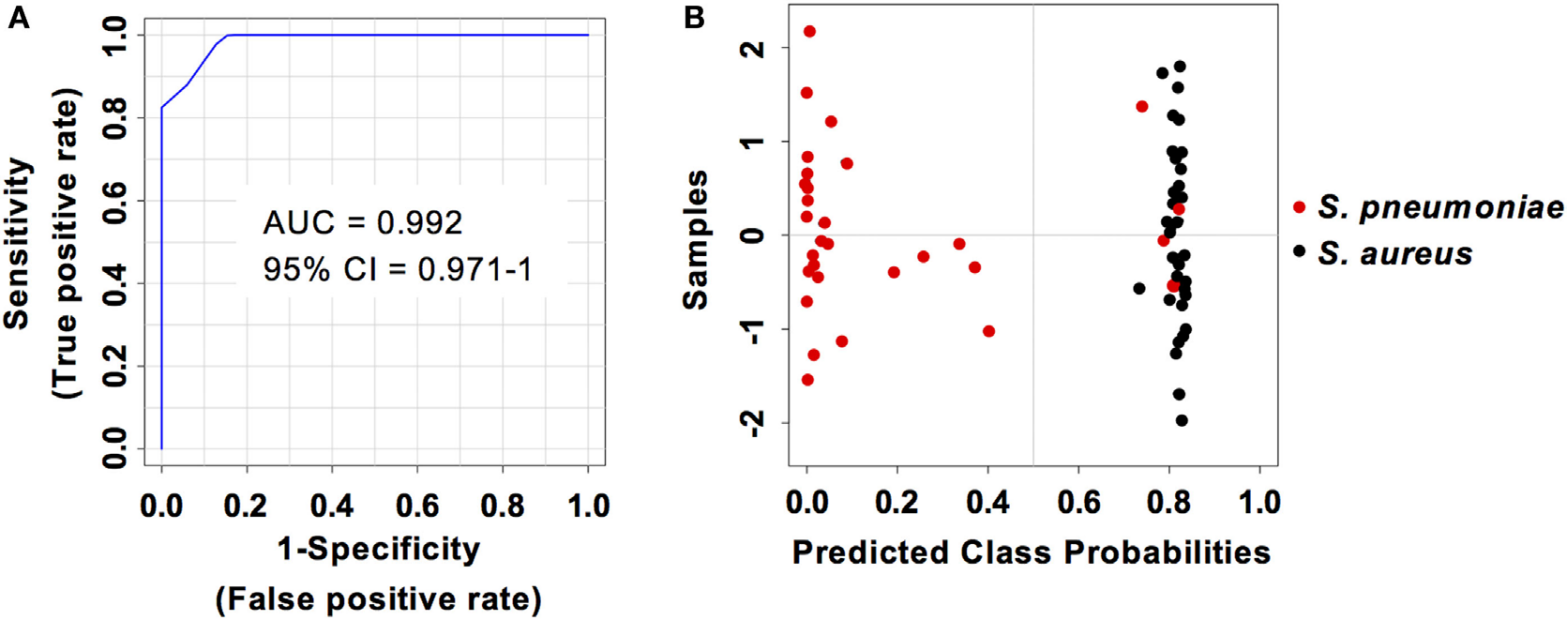
Predictive performance of the predictive model built on the set of the two biomarkers CXCL9 and CXCL10. **(A)** Receiver operating characteristic curve and area under the receiver operating characteristics curve (AUC) value of the predictive model built combining the serum levels of CXCL9 and CXCL10 calculated using Monte Carlo cross-validation. **(B)** Plot of posterior classification probability showing the segregation of *Streptococcus pneumoniae* (red symbols) and *Staphylococcus aureus* (black symbols) infection samples according to the CXCL9/CXCL10 predictive model. Each symbol represents the classification probability that a given infected sample belongs to the *S. pneumoniae* or to the *S. aureus* group. The classification boundary is shown by a dotted line at *x* = 0.5.

Considering that *S. pneumoniae*-infected mice exhibited significantly greater serum levels of IFN-β, IFN-γ, CXCL9, and CXCL10 than *S. aureus*-infected mice, we explored the power of these four biomarkers to discriminate between the two infection groups. Three predictive models comprising 2, 3, or 4 features were built using an SVM. ROC curves and the estimated AUC values for the three predictive models are shown in Figure 6A. The predictive model using two features demonstrated the greater predictive accuracy (94.3%) (Figure 6B). The markers CXCL9 and CXCL10 were selected based on average importance (Figure 6C) for further validation using MCCV analysis. This analysis yielded an AUC of 0.992 (95% CI = 0.971–1) (Figure 7A) and correctly classified 100% of *S. aureus* infection samples and 87% of *S. pneumoniae* samples (Figure 7B).

### Validation of the Prediction Model in an Independent Cohort

**Figure 8:**
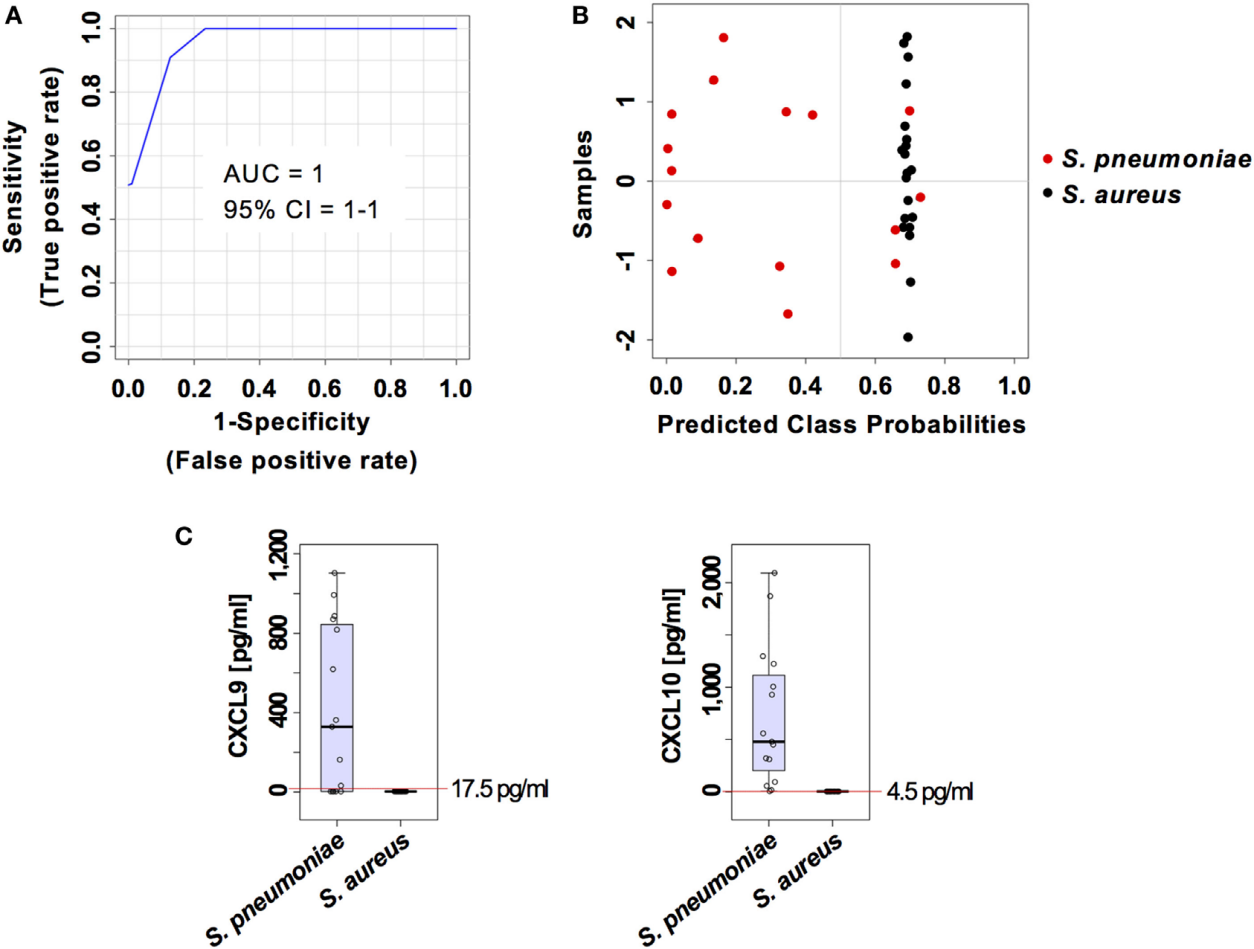
External validation of the predictive model built on the two set biomarkers CXCL9 and CXCL10. **(A)** Receiver operating characteristic curve and area under the receiver operating characteristics curve (AUC) value of the predictive model built combining the serum levels of CXCL9 and CXCL10 calculated using Monte Carlo cross-validation. **(B)** Plot of posterior classification probability showing the segregation of *Streptococcus pneumoniae* (red symbols) and *Staphylococcus aureus* (black symbols) infection samples according to the set of the two biomarkers CXCL9 and CXCL10, using a predictive model in an external cohort of C57BL/6 mice. Each symbol represents the classification probability that a given infected sample belongs to the *S. pneumoniae* or to the *S. aureus* group. The classification boundary is shown by a dotted line at *x* = 0.5. **(C)** Box-and-whisker plots illustrating the concentrations of CXCL9 and CXCL10 in serum of *S. pneumoniae*-infected or *S. aureus*-infection mice. The red line indicates the metabolite concentration cutoff value for sensitivity and specificity of 1 and the corresponding numeric value is included in each graph.

The capacity of the prediction model based on serum levels of CXCL9 and CXCL10 to discriminate between pneumococcal and staphylococcal pneumonia was validated in an independent cohort of 20 mice infected with *S. aureus* and 16 mice infected with *S. pneumoniae* from a different genetic background (C57BL/6) and comprising 50% males and 50% females. The samples from this validation cohort were analyzed under the same conditions than the samples from the training group. The two markers prediction model exhibited an AUC of 1 (95% CI = 1–1) (Figure 8A) and the classification of samples according to infection etiology was very similar to that observed with the training group with 100% correct classification for *S. aureus* infection samples and 12 out of 16 (75%) for *S. pneumoniae* infection samples (Figure 8B). The cutoff serum concentration values for prediction of infection etiology with both sensitivity and specificity of 1 was 17.5 pg/ml for CXCL9 and 4.5 pg/ml for CXCL10 (Figure 8C).

## Discussion

In this study, we performed a genome-wide transcriptional analysis of lung tissue and blood samples of mice with *S. aureus* or *S. pneumoniae* respiratory infection and identified a robust interferon signature consistently associated with pneumococcal pneumonia that was absent in *S. aureus*-infected mice. This interferon signature was manifested by a marked induction of the genes encoding interferons such as IFN-β, IFN-γ, and the interferon-induced mediators such as CXCL9 and CXCL10 in lung tissue and blood cells as well as at the protein level in peripheral blood. The induction of IFN-γ in the lungs in response to *S. pneumoniae* infection has been previously reported (32) and high levels of CXCL9 have been observed in the lungs of mice after intranasal challenge with *S. pneumoniae* (33). Also induction of type I interferon has been shown to be involved in the host response to *S. pneumoniae* in mice (34) and in humans (35). *S. pneumoniae* phagocytosis and recognition of bacterial DNA by cytosolic pattern recognition receptors after translocation from the phagosome into the cytosol seems to be required for IFN-β production by phagocytic cells (36). The translocation of bacterial DNA from the phagosome into the cytosol for induction of IFN-β has been shown to be mediated by pneumolysin, which is a member of the thiol-activated cytolysin family of toxins and an important virulence factor of *S. pneumoniae* (34). Pneumolysin has been also reported to be involved in the induction of IFN-γ by *S. pneumoniae* (35). *S. aureus*, on the other hand, is a poor inducer of interferons (37) and although several studies have reported the capacity of *S. aureus* to activate type I interferon signaling in several types of cells in *in vitro* systems (38, 39), direct induction of interferons by *S. aureus* during *in vivo* infection has not yet been reported. The remarkable resistance of *S. aureus* to degradation within the phagosome due to modifications of the cell wall peptidoglycan that prevents the release of bacterial components necessary to trigger interferon production has been proposed to be responsible for this deficiency (37). This may explain why interferon and interferon-induced mediators were detectable in mice infected with *S. pneumoniae* but not in those infected with *S. aureus* and highlights the potential value of these mediators as biomarkers of pneumococcal pneumonia.

Infection classification models were then built using an SVM to evaluate the potential value of serum levels of these mediators for discriminating between pneumococcal and staphylococcal pneumonia. The infection classifier based on the combination of CXCL9 and CXCL10 serum levels was identified as the best model comprising the minimum set of biomarkers and allowing an accurate infection classification. The robustness of CXCL9 and CXCL10 serum biomarkers in discriminating *S. pneumoniae* from *S. aureus* lung infection was further demonstrated using an independent cohort of mice of a different genetic background and including a combination of males and females. These experimental data altogether identified serum levels of CXCL9 and CXCL10 as powerful diagnostic biomarkers for pneumococcal pneumonia that should advance toward clinical validation in patient cohorts.

Biomarkers are gaining increasing attention within all areas of medicine and infection diseases and have been used already for diagnostic purposes in CAP patients. For example, the levels of procalcitonin (PCT) in blood are elevated in bacterial but not in viral infections (40). Consequently, PCT serum concentration is a suitable biomarker to guide the management of CAP patients since high levels of serum PCT indicate a bacterial rather than a viral etiology (41). However, circulating PCT values cannot discriminate between different bacterial pathogens. Because the results of our study in the murine system indicate that serum levels of CXCL9 and CXCL10 can discriminate between *S. pneumoniae* and *S. aureus* pneumonia, these chemokines may be used as surrogate biomarkers to increase the diagnostic power of PCT and justify future studies to validate their utility as pathogen-specific biomarkers in humans with CAP, and perhaps other invasive infections due to *S. pneumoniae* such as sepsis or CNS infections.

## Ethics Statement

Animal studies were performed in strict accordance with the German animal welfare regulations and the recommendations of the Society for Laboratory Animal Science (GV-Solas). All experiments were approved by the Niedersächsisches Landesamt für Verbraucherschutz und Lebensmittelsicherheit, Oldenburg, Germany (Permit No. 33.19-42502-04-13/1260 and 33.9-42502-04-13/1195).

## Author Contributions

EM and FP conceived the original idea for the study. AS, OG, and EM performed the experiments and analyzed the data. MP performed histological analysis. AS and EM wrote the manuscript. FP contributed to data interpretation and intellectual content and edited the manuscript.

## Conflict of Interest Statement

The authors declare that the research was conducted in the absence of any commercial or financial relationships that could be construed as a potential conflict of interest.
