# Supplementary material for: An Interferon Signature Discriminates Pneumococcal From Staphylococcal Pneumonia
Source: Front Immunol. 2018 Jun 25;9:1424. doi: 10.3389/fimmu.2018.01424 (PMC6026679; doi:10.3389/fimmu.2018.01424)
Supplement: Supplementary file 1 [file Table_1.DOCX]

**Supplementary Table 1****:** Oligonucleotides used in this study for RT-PCR.

| **Primer** | **Orientation** | **Sequence (5´🡪3`)** |
| --- | --- | --- |
| β-Actin | sense | TGGAATCCTGTGGCATCCATGAAAC |
|  | anti-sense | TAAAACGCAGCTCAGTAACAGTCCG |
| Arg1 | sense | ATGGGCAACCTGTGTCCTTT |
|  | anti-sense | TTCCCCAGGGTCTACGTCTC |
| Ccl5 | sense | TATGGCTCGGACACCACTCC |
|  | anti-sense | CACTTGGCGGTTCCTTCGAG |
| Cxcl10 | sense | GCCGTCATTTTCTGCCTCATC |
|  | anti-sense | TAGGCTCGCAGGGATGATTTC |
| Cxcl11 | sense | CGAGTAACGGCTGCGACAAA |
|  | anti-sense | TCACAGTCAGACGTTCCCAG |
| Cxcl3 | sense | ACCCAGACAGAAGTCATAGCCAC |
|  | anti-sense | TGGTGAGGGGCTTCCTCCTT |
| Defb3 | sense | CATTTCTCCTGGTGCTGCTGTC |
|  | anti-sense | CTGCCAATCTGACGAGTGTTG |
| Mx1 | sense | TGACTACCACTGAGATGACCCA |
|  | anti-sense | AGCTGCACTTACTGGTGTCC |
| Mx2 | sense | CCTATTCACCAGGCTCCGAAA |
|  | anti-sense | AGCTGCACTTACTGGTGTCC |
| Ifng | sense  anti-sense | TGAACGCTACACACTGCATCTTGG  CGACTCCTTTTCCGCTTCCTGAG |
