# Supplementary material for: An Interferon Signature Discriminates Pneumococcal From Staphylococcal Pneumonia
Source: Front Immunol. 2018 Jun 25;9:1424. doi: 10.3389/fimmu.2018.01424 (PMC6026679; doi:10.3389/fimmu.2018.01424)
Supplement: Supplementary file 2 [file Presentation_1.PPTX]

## Slide 1
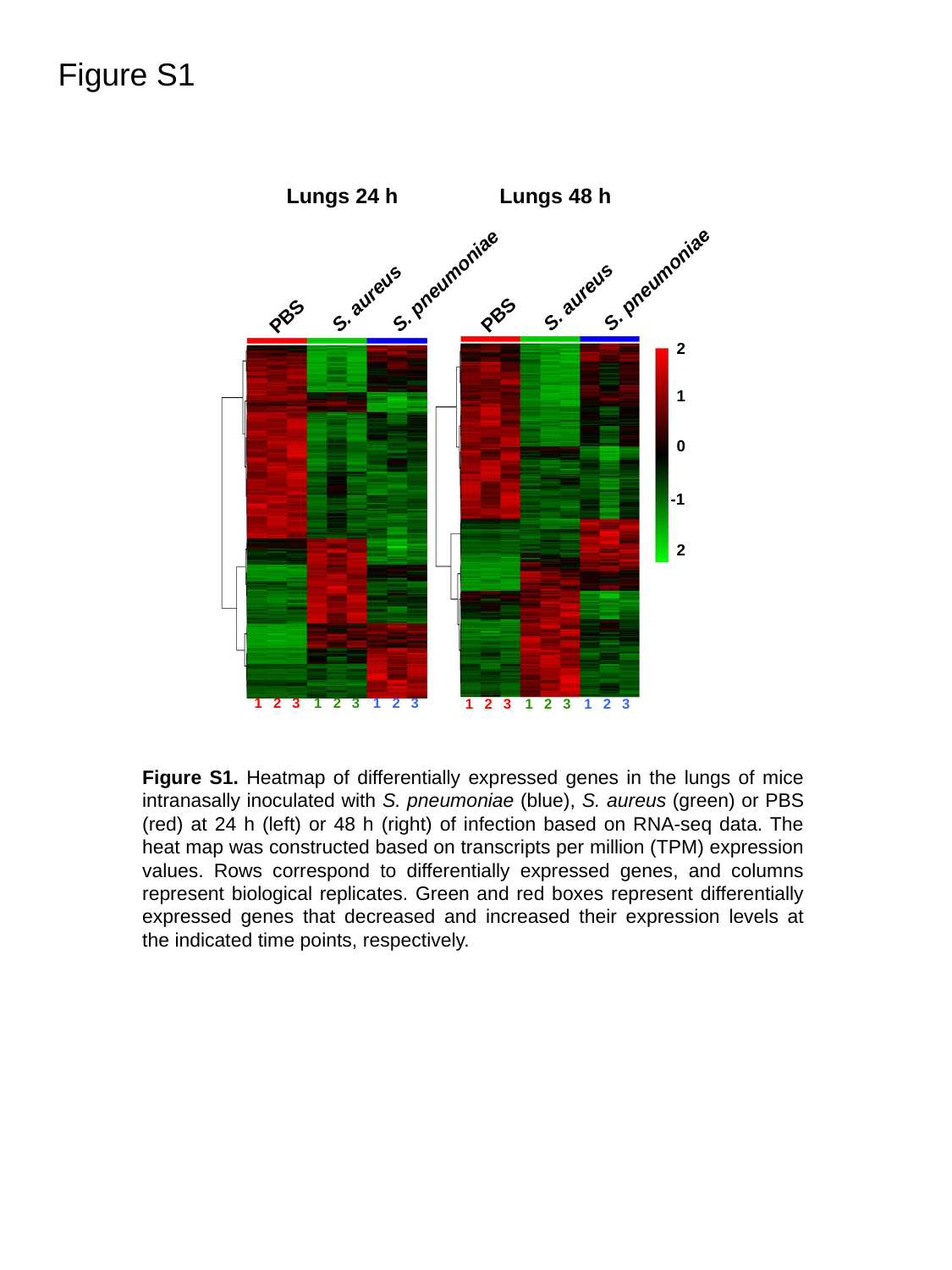

Figure S1
Lungs 24 h
Lungs 48 h
S. pneumoniae
S. pneumoniae
S. aureus
S. aureus
PBS
PBS
2
1
0
-1
2
1
2
3
1
2
3
1
2
3
1
2
3
1
2
3
1
2
3
Figure S1. Heatmap of differentially expressed genes in the lungs of mice intranasally inoculated with S. pneumoniae (blue), S. aureus (green) or PBS (red) at 24 h (left) or 48 h (right) of infection based on RNA-seq data. The heat map was constructed based on transcripts per million (TPM) expression values. Rows correspond to differentially expressed genes, and columns represent biological replicates. Green and red boxes represent differentially expressed genes that decreased and increased their expression levels at the indicated time points, respectively.

## Slide 2
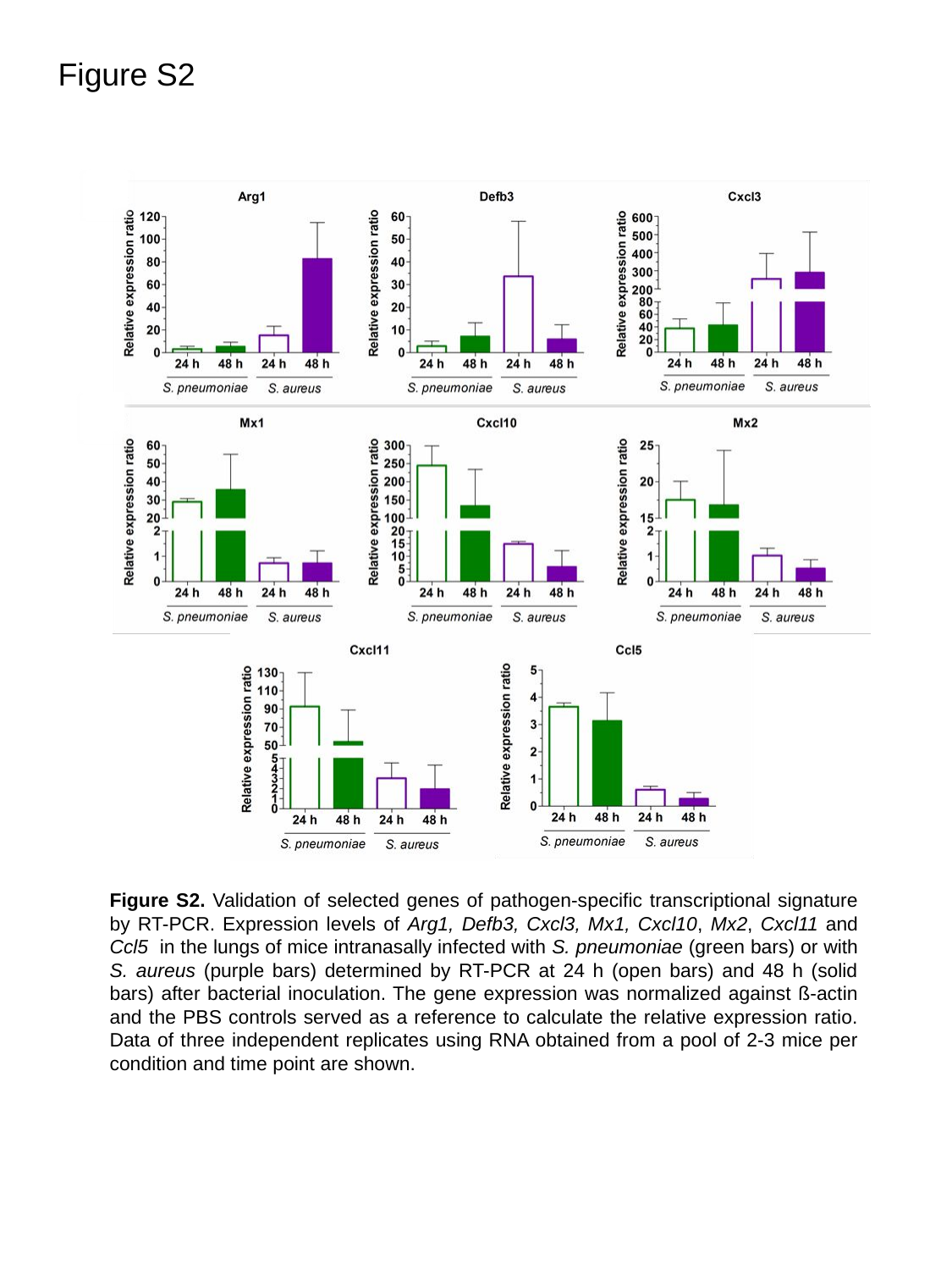

Figure S2
Figure S2. Validation of selected genes of pathogen-specific transcriptional signature by RT-PCR. Expression levels of Arg1, Defb3, Cxcl3, Mx1, Cxcl10, Mx2, Cxcl11 and Ccl5 in the lungs of mice intranasally infected with S. pneumoniae (green bars) or with S. aureus (purple bars) determined by RT-PCR at 24 h (open bars) and 48 h (solid bars) after bacterial inoculation. The gene expression was normalized against ß-actin and the PBS controls served as a reference to calculate the relative expression ratio. Data of three independent replicates using RNA obtained from a pool of 2-3 mice per condition and time point are shown.

## Slide 3
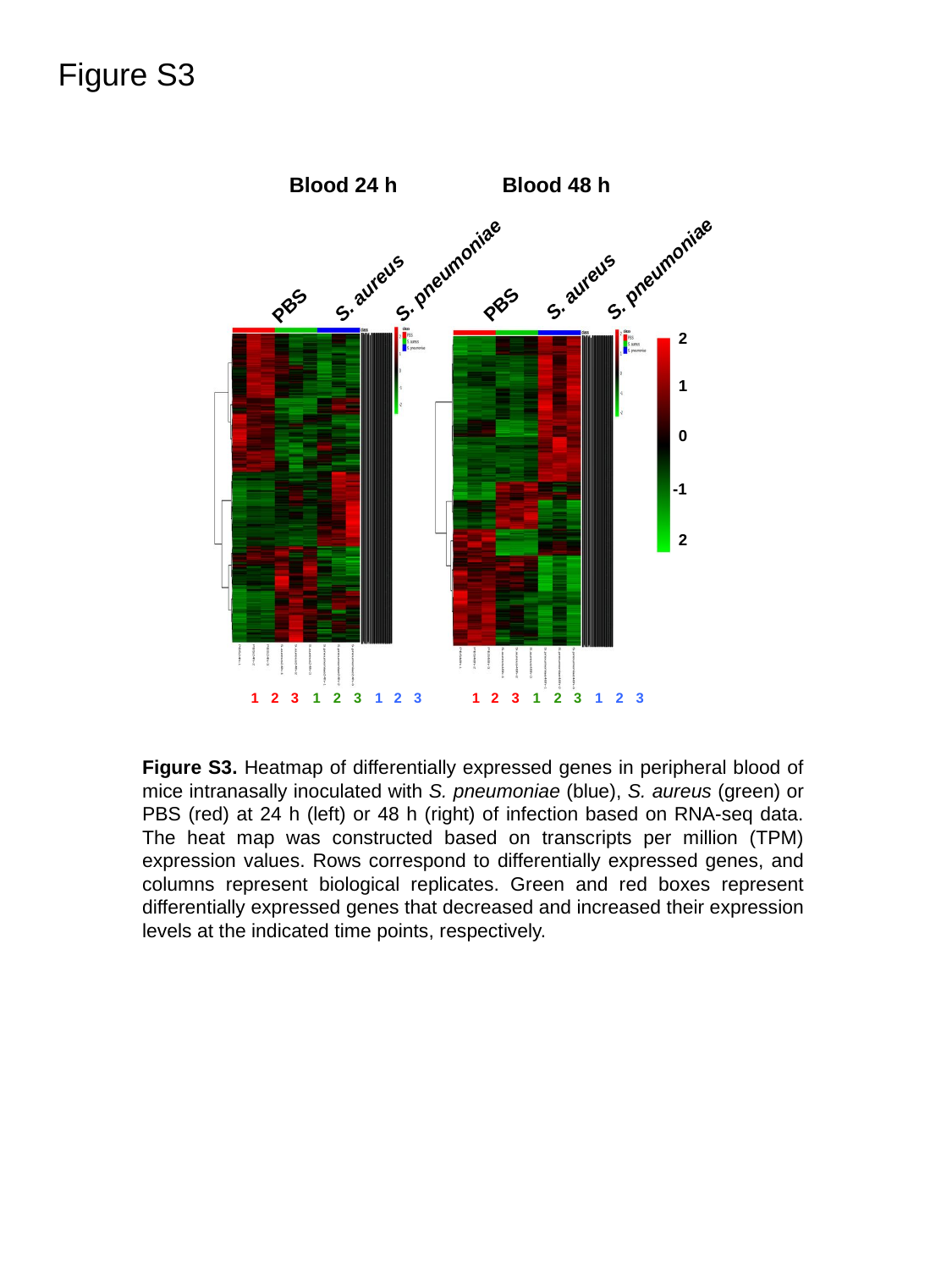

Figure S3
Blood 24 h
Blood 48 h
S. pneumoniae
S. pneumoniae
S. aureus
S. aureus
PBS
PBS
2
1
0
-1
2
1
2
3
1
2
3
1
2
3
1
2
3
1
2
3
1
2
3
Figure S3. Heatmap of differentially expressed genes in peripheral blood of mice intranasally inoculated with S. pneumoniae (blue), S. aureus (green) or PBS (red) at 24 h (left) or 48 h (right) of infection based on RNA-seq data. The heat map was constructed based on transcripts per million (TPM) expression values. Rows correspond to differentially expressed genes, and columns represent biological replicates. Green and red boxes represent differentially expressed genes that decreased and increased their expression levels at the indicated time points, respectively.
